# “*Why Do We Have to Learn This Stuff?*”—A New Genetics for 21st Century Students

**DOI:** 10.1371/journal.pbio.1001356

**Published:** 2012-07-03

**Authors:** Rosemary J. Redfield

**Affiliations:** Department of Zoology, Life Sciences Institute, University of British Columbia, Vancouver, British Columbia, Canada; University of California Berkeley/Joint Genome Institute, United States of America

## Abstract

Our students will go out into an astonishing new world of engineered genes and personal genomics, so why is the standard genetics syllabus stuck in the 1950s?

Several years ago, our biology program decided to develop a new second-year “Fundamentals of Genetics” course to replace the third-year course that was our legacy from David Suzuki and Tony Griffiths. Although our new syllabus radically altered how the core concepts are taught, I now think the changes were much too conservative because we'd ignored how drastically the role of genetics has changed. Below I first describe the problems we originally identified and how we addressed them, and then consider the bigger problem of moving introductory genetics courses into the 21st century.

## The Canon

Our old course followed the canonical textbook structure, using genetic analysis (see Box 1 for Glossary) to teach the principles of transmission genetics, with the history of genetics providing the organizing framework (see, for example, [1],[2]). It began with Mendel's experiments and the principles they revealed—phenotypes are determined by genes whose alleles pass unchanged through the generations; alleles are dominant or recessive; pairs of alleles separate into gametes and form new combinations in each new generation. Next came the evidence that genes are on chromosomes, followed by the light-microscopy view of chromosome behaviour in meiosis. Analysis of genetic crosses then gradually revealed all the standard concepts—linkage and crossing over, gene mapping, sex linkage, epistasis, chromosome changes. Some instructors supplemented these topics with a smattering of molecular genetics, but serious treatment was left to a later course using the second half of the textbook. The term finished with a few lectures on the basic theories of population and quantitative genetics as established in the mid-20th century.

**Box 1. Glossary**

**Epigenetic:** heritable differences due to reversible modification of DNA rather than to changes in DNA sequence.

**Epistasis:** change in phenotype caused when different alleles of one gene alter the effects of alleles of another gene.

**Exome:** the expressed part of the genome, all the exon sequences.

**Genetic analysis**: using the phenotypic outcomes of genetic crosses to make inferences about inheritance and gene function.

**Homologous chromosomes:** chromosomes carrying different versions of the same genes in the same order. Usually these DNA sequences differ by only about 0.1%.

**Haplosufficiency:** a condition where having one functional and one nonfunctional version of a gene provides sufficient gene activity to give a normal phenotype.

**Inversion-heterozygote:** A situation where one of a pair of homologous chromosomes has an inversion that reverses the order of genes in one segment. The two chromosomes must contort to pair the inverted part with its homologous sequences.

**Quantitative genetics:** Analysis of the contributions of multiple, often hypothetical, genes to a single phenotype.

**Tetrad analysis:** using a micromanipulator to separate the four spores or gametes produced by a single meiosis and then analyzing their genotypes.

**Three-factor cross:** a cross whose partners differ at three different positions on a single chromosome; typically used to map the relative locations of the genes.

This historical presentation has a long-established rationale [3]. Having students follow in the intellectual footsteps of the great geneticists should make the concepts easier to learn because it mirrors the questions students will naturally ask. Students are also expected to gain much-needed practice in scientific thinking and a better appreciation of the nature of scientific knowledge because each new principle is necessitated by experimental evidence. And finally, seeing how genetic analysis has revealed the mechanisms of inheritance should show students its value in dissecting other biological phenomena.

Unfortunately, this wasn't working as planned; although students learned to solve our genetic analysis problems, their ability to think scientifically didn't noticeably improve and they didn't seem to understand much genetics. For example, although most students' test results showed that they could predict and interpret phenotypes in crosses, conversations at office hours and tutorials revealed that they had only very unconventional ideas about how gene products interact to determine phenotype (see Box 2: The Dominance Problem). And although they could reproduce the stages of meiosis, map genes in three-factor crosses, and diagram meiotic recombination in complex inversion-heterozygotes, most had no idea how or why homologous chromosomes pair and recombine. Although not all instructors report these problems, similar persistent misconceptions have been described by Smith and Knight [4]).

**Box 2. The Dominance Problem**

Mendel's concept of dominance leaves students deeply confused about both its meaning and its causes. Most students mistakenly believe that alleles are intrinsically either dominant or recessive, as did Mendel. But dominance is a relationship between alleles—one allele is dominant to another if its homozygous phenotype is also seen in the heterozygote. Mendel's error was reasonable because he had limited his analyses to allele pairs, but the convenient but erroneous *A*/*a* representation he introduced has propagated his misconception through textbooks and homework problems.

Students also mistakenly think that dominant/recessive relationships are the norm. This is largely because almost all the alleles they see in their genetics course are presented in dominant/recessive pairs, with alternatives presented only as variants of or exceptions to dominance (“codominance” and “incomplete dominance”). This problem also has been reinforced by generations of geneticists, who, like Mendel, have preferred to work with alleles showing clear-cut phenotypic differences. This issue is becoming increasingly important as we uncover the genetic underpinnings of natural phenotypic variation, very little of which shows classical dominance.

Finally, even though most modern textbooks explain that dominance is usually caused by haplosufficiency, informal questioning of students in our upper-level genetics course reveals that most still have no idea what makes one allele dominant to another. They have a hard time even understanding the question, and when pressed they typically speculate that dominant alleles must actively turn off their recessive partners, perhaps by acting as repressors or via epigenetic effects.

The committee responsible for developing our new course thought that the historical approach was making genetic analysis harder for our students to learn, not easier. Understanding even the simplest cross requires combining inferences about two complex processes—how genetic elements are inherited through meiosis and mating, and how these elements act and interact to cause the phenotypic differences seen in the parents and the progeny. Pioneer geneticists treated these processes as “black boxes” whose rules they deduced, but our students appeared to avoid this challenge by simply memorizing the rules and problem-solving rubrics that well-meaning instructors provided. Compounding the difficulty, classic experiments often require students to also understand complex experimental details unrelated to the principle being taught (e.g., the use of pneumococcal transformation to show that DNA is the genetic material). There was no time or resources for a formal study (the first validated tools for assessing genetics learning were just becoming available [5],[6]), so we went ahead and put our best ideas into the new course.

Our new syllabus addresses these problems by dispensing with the historical approach and by deferring analysis of crosses until after the underlying processes have been explicated. Thus, our course begins not with Mendel but with two independent blocks, first a three-week block on gene function (how genotype determines phenotype), and then a two-week block on inheritance. Our second-year students come to us knowing the basics of molecular biology, but they haven't thought about how differences in DNA cause differences in phenotypes, especially not in diploids (hence their deep confusion about dominance). Block 1 teaches how diploid phenotypes arise from homozygous and heterozygous differences in genes with various simple and/or interacting functions. Students first encounter dominance and epistasis here, not as abstractions but as predictable consequences of the metabolic and regulatory interactions between functional and non-functional proteins. In Block 2, students learn to follow DNA sequence differences and named alleles through meiosis and mating, free from the confusion of phenotypes. Meiosis is the biggest obstacle for most genetics students, and this block emphasizes its function and molecular mechanism. Instead of memorizing the “stages” seen under a light microscope, students consider first the problems mitosis and meiosis must solve (how to get the right chromosomes into the daughter cells) and then their molecular solutions (cohesin, separase, spindle fiber tension, and especially the role that sequence recognition by invading DNA strands plays in homolog pairing and crossing over). The effects of independent chromosome assortment and of crossing-over are explored by first explicitly working out the genotypes of the gametes produced by meiosis, and then using Punnett squares to represent how gametes come together randomly in mating. Once these blocks have solidified the fundamental principles of phenotypes and inheritance, genetic analysis is used to teach the more complex concepts.

## The Canon Is Past Its Sell-By Date

We were all proud of the new course's syllabus, and it was only when I taught the first section last year that I realized how obsolete the contents are. I'd introduced a brief “Genetics in the News” segment at the start of each class; my intent was to show students the relevance of what I was teaching them, but the torrent of genetics news I was sorting through instead showed me its irrelevance.

Our goal in designing the course had been to make students competent in the standard principles of transmission genetics, but we had totally failed to consider whether this is really what our students need to know. In fact, we'd been failing to do this for many years—complaining about students forgetting everything we'd taught when the course ended, but never once thinking that this might be (i) our fault for teaching them material they would never use and (ii) correctable by making the course content more relevant. Fifty years ago this wouldn't have been a big oversight, but now genes are everywhere, sometimes trivially (“the DNA of Toyota”), but more often with serious implications for personal and public life.

Perhaps the biggest change is the rise of direct-to-consumer genetic and genomics services. (These services aren't only for people: $69.99 plus a cheek swab will tell you the ancestry of your mutt, and $19.50 plus a feather will tell you whether your parrot is Polly or Paul.) As of October 2011, more than 125,000 customers of 23andMe have signed up for personal genotyping at a million SNP positions and access to 23andMe's excellent interpretive resources. Direct-to-consumer exome sequencing is already available as a pilot venture ($999 for 70-fold coverage), and the promised $1000 genome sequence will be cheaper than most MRI scans. By the time our students become parents, standard obstetrics packages may include sequencing the baby's genome.

A good place to see these and other changes is headline news—Box 3 gives some high-ranking hits from a recent Google News search for “genetics”. These raise complex questions, both personal and societal, that our students will need to answer. Is genetic testing a wise thing to do? Is it a sound financial investment? Should I have full access to my genetic information? Should my insurer and my employer? Should athletes be tested for genetic modifications (“gene doping”)? Is it ethical to DNA-fingerprint all convicted criminals? All suspects? Did my genes make me gay? Are genetically modified foods safe? Are cloned animals ethical? How different are human races, and how different are we all from chimpanzees and gorillas?

**Box 3. Headlines from a Google News Search on March 19, 2012**

- First Complete Full Genetic Map of Promising Energy Crop (*Mapping the genome of Miscanthus.*)
- New Genetic Test Predicts Better Egg Production for Women with Poor Ovarian Reserve (*Heterozygosity for the “fragile-X” gene improves fertility.*)
- DNA Electronics Partners with geneOnyx to Offer Genalysis (*Personal DNA testing for the beauty industry.*)
- Geneticist's Personalized Medicine Study Focuses on Himself (*How Mike Snyder predicted and tracked his own diabetes.*)
- Genetic Tests to Generate 25 Billion a Year, UnitedHealth Says (*Should you invest now?*)
- International Conference on Consanguineous Marriage Continues (*A Middle-East country hopes to reduce genetic problems caused by inbreeding.*)
- Prehistoric Iceman's DNA Reveals Startling Secrets (*He was just like us!*)
- Genetic Testing's Growth Raises Legal and Ethical Concerns (*Will these outweigh the expected savings in health-care costs?*)
- Genetics Not Education Drives Investor Behaviour (*Heritability is 50% in a identical-twin study*.)
- Gorillas More Related to People Than Thought, Genome Says (*15% of gorilla genes are closer to humans than to chimps.*)

The nature of genetics research has changed too. For our own and other species, emphasis has shifted from laboratory mutations to natural genetic and phenotypic variation. Humans are rapidly becoming the best-understood genetic system; there are many more complete genome sequences for humans than for any other species, and more detailed surveys of natural variation [7],[8]. This natural variation used to be largely out of reach, only investigated using the abstractions of classical quantitative genetics, but genome-wide association studies are now able to find genes that affect just about any phenotype. One consequence is that genes are now studied in the context of populations, since crosses can't distinguish the small phenotypic effects typical of natural variation. Finally, evolution is now explicitly intertwined with genetics at every point, both firmly embedded in differences between DNA sequences. Sequence conservation tells us which parts of genes are essential for function, and analysis of gene families tells us how functions change.

Changes in the student population have made the canon even less relevant. Genetics used to be an advanced elective for intellectually ambitious students, but most biology programs now require it of everyone. Many of these students will never take another genetics course, much less engage in genetics research. On the other hand, students destined for professional programs in the health sciences need much more genetics than they used to, since they'll increasingly be requesting genetic tests and explaining the results to their patients. Fortunately, the genetics they'll need is just a slightly more sophisticated version of what the others will need in their daily lives—a solid understanding of how genes influence phenotypes, of natural genetic variation, and of the mechanism of heredity.

Instructors are certainly not unaware of these changes, and many have modified what they teach to include as much new material as possible. But patching new material onto our outdated genetics canon fails to address its fundamental obsolescence. It's time to make a fresh start.

## A Clean Break with the Canon

Box 4 gives a suggested syllabus for a 21st century genetics course. It begins with a human focus, introducing personal genomics and our natural genetic variation. Students then learn about the underlying molecular explanations—how differences in DNA sequences arise and evolve, and how they cause differences in phenotype—followed by how genetic differences are inherited and recombined. With this under their belts, students are ready for a taste of genetic analysis, maybe just enough to whet their appetites for an advanced course on genetic methods. The course then returns to natural genetic variation, now considering how it can be studied, and how to interpret the results. Nothing is taught as a black box—everything is presented in the context of its molecular underpinnings.

**Box 4. Suggested Syllabus for a 21st Century Genetics Course**

- Personal genomics
- Natural genetic variation in populations (humans and others)
- Structure and function of genes and chromosomes
- Genetic variation arises by mutation
- Genetic variation and evolution (selection for function, phylogeny, homologs, gene families)
- How genes affect phenotypes: pathways, regulatory interactions, heterozygosity, dominance effects (*several classes*)
- Genetic variation also arises by chromosome reassortment and homologous recombination
- Mitosis and meiosis: mechanisms and genetic consequences (*several classes*)
- Mating: mechanisms and genetic consequences
- Linkage and sex linkage
- Genetic analysis: investigating gene action using inheritance of simple (“Mendelian”) alleles and phenotypes in crosses and pedigrees (*several classes*)
- Organelle genetics
- Epigenetic inheritance
- Genome structure, function and evolution; causes and consequences of chromosomal changes (*several classes*)
- Phenotypic effects of natural genetic differences, heritability
- Genome-wide association studies and related studies linking genes to phenotypes (*several classes*)
- Genetics of cancer; inheritance of alleles affecting risk

This radical a change will encounter lots of obstacles. For many geneticists the most upsetting change will be the demotion of genetic analysis from its reigning place in the curriculum. Genetic analysis used to be the most powerful tool for understanding how organisms work, and thus the best skill we could give our students, but its research role has been largely supplanted by molecular methods. Cuts to genetic analysis also threaten the problem-based learning that has been a hallmark of genetics courses. Genetics instructors have all devoted time to developing problems that replicate those arising in real genetics research labs, and a major feature in textbook choice is the quantity and quality of the end-of-chapter problems.

Other cuts will be less traumatic. Our students will probably never need to do a 3-factor cross, except maybe in an outdated genetics laboratory course, nor to analyze phenotypic ratios of progeny, once “one of the pillars of genetics” [1]. There's also little justification for retaining haploid genetics, fungal genetics, tetrad analysis, and classical somatic-cell genetics in an introductory genetics course. Classical bacterial genetics (conjugation, transduction, transformation) should go too—I'm a bacterial geneticist, so trust me on this one.

One of the biggest obstacles is purely practical—the lack of any suitable textbook. Although most of the topics I would introduce are at least touched on by current textbooks, the material is not very useful because it's in advanced chapters, not integrated into the core material. Textbook publishers are very conservative, and even books with an ostensibly molecular focus usually leave the canon intact. Online resources may be able to fill the gap, but finding and modifying them will still be a lot of work.

As a first step, geneticists need to step back from the current curriculum and decide what 21st century students really need to know about genes and inheritance. These decisions should be based on how students will use what they learn, and not on what we as geneticists value. Then we can develop specific learning goals—lists of skills we want students to gain from our teaching. Only then will we be ready to develop a syllabus, and to create the textbooks, assessment tools, and validation tools we'll need. At the same time, we should be promoting parallel changes at earlier levels; the brief time high school and first-year university students devote to genetics shouldn't be wasted on Mendel's laws and Punnett squares.

I expect that just reading this article will have raised the hackles of many readers whose favourite topics I would cut. Procrastination is attractive—if we wait long enough, maybe the pace of change will slow, the issues will become clearer, and traditionalist colleagues will retire. As long as we remain comfortable with teaching largely irrelevant material, we don't have to worry about changing it. But if we want to make the genetics we teach genuinely useful to our students, we need to start the process now. 

*To get the discussion going, please consider describing your experiences and suggestions in the Comments section. (Editor's note: Click on the “Comments” tab under the title of the article.)*
